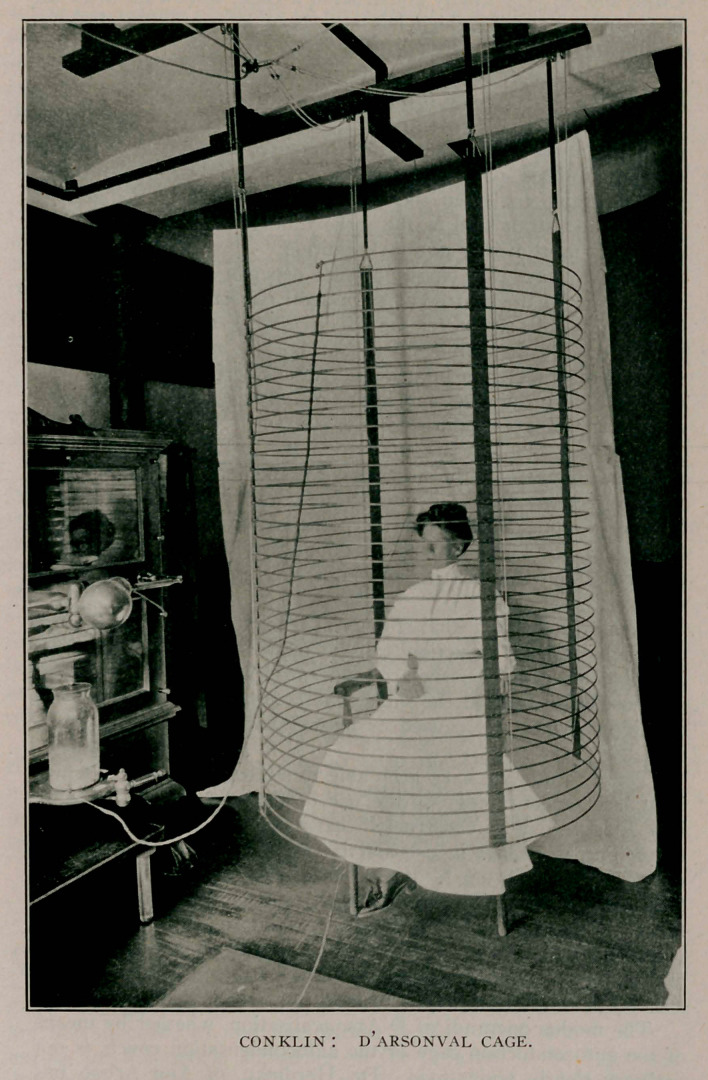# The Effect of High Frequency Currents upon Blood Pressure. Demonstration of the D’Arsonval Cage1Read at the forty-first annual meeting of the Medical Association of Central New York, held at Buffalo. October 27, 1908.

**Published:** 1909-06

**Authors:** W. L. Conklin

**Affiliations:** Dansville N. Y.


### The Effect of High Frequency Currents upon Blood Pressure. Demonstration of the D'Arsonval Cage.[1]

By W. L. CONKLIN, M. D., Dansville N. Y.

LAST summer I had under my care a patient sixty-three years of age who was suffering from that not infrequent pathological combination, damaged kidneys, heart and arteries. When she came to the Jackson Health Resort in June, she was recovering from a sharp attack of bronchitis, dyspnea being marked on the least exertion, and with a blood pressure of 225 the outlook was not very good. With absolute rest in bed for some time, limited diet, a long course of Schott baths, massage, and the usual medication she did very well and returned to her home in November much improved. Last spring I learned that after some slight overexertion she became suddenly worse and died in a few days.

My patient was extremely fond of music and in many other ways well equipped for years more of useful and happy life. I have gone thus into detail regarding her case because it is typical of a large class of patients suffering from this pathological trinity so likely to terminate life years before its period of usefulness would otherwise be ended.

No doubt great advance has been made in the management of this condition, resulting from a better knowledge of its causes, but there is still room for improvement, and it is for this reason that I call your attention to a method of treating high arterial tension which, though not as yet much in vogue, promises to be of value.

During the period the patient to whom I have referred remained at the Health Resort she called my attention to an article on the treatment of hypertension, which appeared in *L'Illustration,* June 22, 1907, and later made a translation for me. The article gave an account of the D'Arsonval cage treatment of high tension and unlike many effusions on medical subjects in the papers and magazines it was written with an evident consideration for the truth and not primarily to produce a sensation. I will quote from it the accurate and quaintly put definition of high blood pressure:

> Hypertension is not in itself a malady, it is an indication, a warning, a state coming before, which it is well to recognise in time and interpret in order to combat the evil with which it will be followed if one does not administer a remedy.

After reading this article and a similar one in the *Scientific American,* and reflecting on my patient's expression of regret that the method of treatment which they described had been but little

1. Read at the forty-first annual meeting of the Medical Association of Central New York, held at Buffalo. October 27, 1908.

CONKLIN: D'ARSONVAL CAGE.

used in this country, I sought the aid of our superintendent, Mr. Croll, and he very kindly constructed a cage, somewhat after the D'Arsonval pattern, which has now been in use in the Jackson Health Resort since March 1, 1903.

We have all experienced difficulty in securing desired results in the treatment by the ordinary means of the class of cases in which high arterial tension occurs. We have all felt, too, I have no doubt, that even though this increased tension and arterial degeneration are only symptoms of trouble elsewhere, still it is very desirable to lessen the tension and thus save the arteries from undue strain and at the same time give them a chance to get into a more nearly normal condition.

Janeway says that blood pressure depends upon four factors: (1) energy of the heart; (2) peripheral resistance; (3) elasticity of arterial walls; (4) volume of circulating blood. There can be no question that while the blood pressure as measured by the sphygmomanometer is influenced by the degree of sclerosis, the reverse is also the case, and high tension has a tendency to produce or increase a conditon of sclerosis.

Osler says, "There are four great factors in the causation of arteriosclerosis: (a) the normal wear and tear of life; (b) the acute infections; (c) the intoxications and (d) those combinations of circumstances which keep the blood tension high." He further says, "There can be no doubt that in many individuals the rise of blood pressure antedates the appearance of arteriosclerosis."

It is evident, then, that if arterial tension can be reduced and especially if that reduction can be made to a greater or less degree permanent, then we are rendering less potent one cause of arteriosclerosis, and at the same time combatting other abnormal conditions which are caused or aggravated by high blood pressure.

These results we have been able to secure, to a degree which is at least encouraging, by the use of the cage which I exhibit here today. It is so constructed that it may be drawn up toward the ceiling when not in use and is simply a large solenoid consisting of 32 coils of ¼ inch brass-plated rod. The coils are two inches apart and each is 12 feet long. This solenoid is attached at the upper and lower extremities of the spiral to two pairs of Leyden jars which are, in turn, attached to the poles of a static machine. If desired, the lower attachment may be with a metal plate underneath the platform on which the patient sits.

The modus operandi of D'Arsonvalisation, whether by means of the autoconduction cage or the autocondensation couch, is not yet very clearly understood. Dr. Herdman, of Ann Arbor, has made experiments with animals which throw some light upon the subject and it is at least proven that one effect is to render more active the various processes of metabolism and elimination. For example, an increased elimination of urea is one of the results quite uniformly noticed. It may be that we shall find here a valuable method of treating that large class of cases of mental depression and even insanity due to faulty elimination. Thus far, at the Jackson Health Resort, the cage has been used mainly in the treatment of cases showing a blood pressure above normal.

The patient remains within the cage from fifteen to twenty minutes and is surrounded by an electrical atmosphere, so to speak, of high frequency and high potential; constituting the autoconduction treatment first advocated by D'Arsonval. The reduction of blood pressure in a fifteen-minute treatment is ordinarily from 4 to 10 mm. though as great a reduction as 15 mm. has been noted. The treatments are given from two to four times each week. The blood pressure was 170 in one case when the treatments were begun, April 6, and gradually fell to 135, where it has remained without change since the treatments were discontinued four weeks ago.

In another case there was an average drop of 8.7 mm. in ten treatments. Highest blood pressure noted 170, lowest 142. This lady had suffered for many years with periodic attacks of mental depression which seemed to be due to faulty elimination. Decided improvement in her condition was noticed after a few cage treatments had been given. One patient in whom the highest pressure noted was 146 mm. remains at about 125 mm. with one fifteen-minute treatment each week. The treatments have just been begun in a case in which the pressure is about 260. The arteries are very hard and thus far results have not been well marked.

I will not weary you with further detail but will only say in closing that while my experience with the D'Arsonval cage is not yet extensive I am strongly inclined to the belief that we have in it a valuable aid in the treatment of the various pathological conditions in which high blood pressure is an important factor.

## Figures and Tables

**Figure f1:**